# Medicinal Uses of Ptelea Trifoliata

**Published:** 1866-05

**Authors:** O. F. Potter

**Affiliations:** St. Louis, Mo.


### MEDICINAL USES OF PTELEA TRIFOLIATA.

By O. F. POTTER, M.D., St. Louis, Mo.

I wish to call the attention of physicians to a plant, the medical virtues of which I have been familiar with for some years, and from personal experience would recommend it to the favorable notice of the profession.

The plant is known as the ptelea trifoliata, or, commonly, as the wafer-ash or wingseed, a species of so-called swamp dogwood, and is of the natural order Xanthoxylacæ.

It is a shrub cf from six to eight feet in height. The leaves are trifoliate and marked with pellucid dots. The leaflets are sessile, ovate, short, accuminate, downy beneath, lateral ones inequilateral, terminal ones cunate at base, from three to four inches long by one to one and three-fourths inches wide. The flowers are polygamous, nearly one-half inch in diameter, of a greenish-white color, and of a disagreeable odor. Stamens mostly four with style short; fruit, a two-celled samara, nearly an inch in diameter, winged all round, nearly orbicular. It flowers in June. It is common to this country, growing more abundantly west of the Alleghanies, in shady, moist, and rocky places generally at the edge of woods. The bark of the root possesses its peculiar medicinal properties, which it yields to boiling water, but alcohol is its best solvent. The bark, when dried, is of a light brownish-yellow color, comes in cylindrical rolls of quills a line or two in thickness, and from one to several inches in length; is irregularly wrinkled externally, and is covered with a thin epidermis; internally it is of a yellowishwhite, but becomes darker on exposure. It has a peculiar, rather aromatic smell, and a bitter, pungent, and rather acrid taste, yet nothing disagreeable; the pungency is persistent, which is owing to the oil which it contains.

I have been using it as a tonic to follow the use of quinine in all grades of fevers, also in cases of general debility connected with gastro-enteric irritation. It is mild, unirritating, having a soothing influence on the stomach, promoting digestion. It promotes the appetite, enabling the stomach to endure suitable nourishment, and favors the early re-establishment of the digestive functions, and will be tolerated by the stomach when almost all other tonic or stimulant remedies are rejected. I have found it especially useful in cases of debility following a low grade of fever, also with females after confinement, or where the menstrual functions are deranged, frequently by sustaining the digestive and secretive functions, regulating the menstrual flow; also, as a sustaining and strengthening stimulant in debility connected with or following wasting ulcers or scrofulous sores.

I have been in the habit of using it in the form of a tincture, made by putting six ounces of the bark and one-half ounce of ginger to two quarts of whisky; the dose from one to two tablespoonsful three times a day for an adult.

I feel assured, from over ten years' experience in using it, that it will be found a most valuable and reliable remedy. It has been used occasionally by the so-called eclectic physicians, and also by the negroes of the South, who call it the scrofula root, from its usefulness in sustaining the system when debilitated by that so common disease amongst them. The old French inhabitants near St. Louis also used it many years ago as a cure for the intermittent fevers of the country, long before quinine was known. When used for a great length of time, or in very large doses, it occasionally, in some persons, occasions an erysipelatous inflammation in the surface, which, however, only lasts for a short time if its use is persevered in, and no ill effects follow it.—*N. Y. Med. Jour., Dec., 1865.*—*St. Louis Medical Reporter.*

